# Total fecal microbiota transplantation alleviates high-fat diet-induced steatohepatitis in mice via beneficial regulation of gut microbiota

**DOI:** 10.1038/s41598-017-01751-y

**Published:** 2017-05-08

**Authors:** Da Zhou, Qin Pan, Feng Shen, Hai-xia Cao, Wen-jin Ding, Yuan-wen Chen, Jian-gao Fan

**Affiliations:** Center for Fatty Liver, Department of Gastroenterology, Xinhua Hospital Affiliated to Shanghai Jiao Tong University School of Medicine, Shanghai, 200092 China

## Abstract

Non-alcoholic steatohepatitis (NASH) is an epidemic metabolic disease with limited therapeutic strategies. Cumulative data support the pivotal role of gut microbiota in NASH. Here, we investigated the hypothesis regarding whether fecal microbiota transplantation (FMT) is effective in attenuating high-fat diet (HFD)-induced steatohepatitis in mice. Mice were randomized into control, HFD and HFD + FMT groups. After an 8-week HFD, FMT treatment was initiated and carried out for 8 weeks. The gut microbiota structure, butyrate concentrations of the cecal content, liver pathology and intrahepatic lipid and cytokines were examined. Our results showed that after FMT, the gut microbiota disturbance was corrected in HFD-fed mice with elevated abundances of the beneficial bacteria *Christensenellaceae* and *Lactobacillus*. FMT also increased butyrate concentrations of the cecal content and the intestinal tight junction protein ZO-1, resulting in relief of endotoxima in HFD-fed mice. Steatohepatitis was alleviated after FMT, as indicated by a significant decrease in intrahepatic lipid accumulation (reduced Oli-red staining, decreased intrahepatic triglyceride and cholesterol), intrahepatic pro-inflammatory cytokines, and the NAS score. Accordingly, intrahepatic IFN-γ and IL-17 were decreased, but Foxp3, IL-4 and IL-22 were increased after FMT intervention. These data indicate that FMT attenuated HFD-induced steatohepatitis in mice via a beneficial effect on the gut microbiota.

## Introduction

A global non-alcoholic steatohepatitis (NASH) crisis, which was provoked by an obesity epidemic, has emerged and lacks effective therapeutic strategies^1^. The pathophysiology of NASH is multifactorial and has strong genetic and environmental contributions. Cumulative data demonstrates that the dysbiosis of the gut microbiota is greatly associated with the onset and progression of NASH^2^. Structural disruption and the associated inflammation of the gut microbiota are considered important etiological factors, which can result in immunologic dissonance and disturbance of metabolites produced by the gut microbiota^3, 4^. A better understanding of the gut microbiota and its metabolites in liver diseases might form the basis for novel therapies. ‘Gut microbiota-targeted’ interventions might be an effective approach to treat metabolic disorders, including NASH. Increasing evidence demonstrates that fecal microbiota transplantation (FMT), which is a new and underexplored method to alter the gastrointestinal microbiota, is definitely effective for the treatment of patients with chronic gastrointestinal infections and inflammatory bowel diseases. However, the therapeutic potential of FMT-based intervention for NASH and the further mechanisms associated with FMT have been poorly investigated to date, even at animal models^5, 6^.

Notably, the bias of liver-infiltrating T cells along with their secreted cytokines plays a pivotal role in the inflammation and progression of NASH^7^. Meanwhile, the gut microbiota plays an essential role in maintaining homeostasis of the adaptive immune system and regulates T cell functions, which may be partially attributed to metabolites such as short-chain fatty acids (SCFAs)^8–10^. Thus, we conducted this exploration and hypothesized that FMT would improve the gastrointestinal microbiota ecosystems, regulate gut metabolites such as short-chain fatty acids and correct the imbalance of the liver immune system, leading to attenuation of high-fat diet (HFD) -induced steatohepatitis in mice.

## Materials and Methods

### Animal experiments

Specific pathogen free (SPF) male C57BL/6 mice (SLAC laboratory animal co., LTD, Shanghai, China) were housed in high-efficiency, particulate air-filtered cages with sterilized bedding and fed autoclaved chow and water *ad libitum*. All animal experiments were approved by the Institutional Animal Care and Use Committee of Xinhua hospital, which is affiliated with the Shanghai Jiao Tong University School of Medicine, and all experiments were conducted in accordance with the National Research Council Guide for Care and Use of Laboratory Animals.

Mice were randomly divided into three groups (12 mice per group). The control group was fed with standard chow for 16 weeks. The HFD group was fed a HFD (88% standard diet, 10% lard and 2% cholesterol) for 16 weeks. The HFD + FMT group was fed with a HFD for 16 weeks and treated with FMT for the latter 8 weeks. All mice were given a combination of penicillin (2000U/ml) and streptomycin (2 mg/ml) (Sigma Aldrich, USA) in their drinking water for 3 days to remove indigenous gut microorganisms. After this treatment, 200 mg of fresh stool was collected from the control group immediately upon defecation and was resuspended in 5 ml of normal saline, vortexed for 3 min and allowed to settle by gravity for 2 min, and the fresh stool was collected every day. Transplant into recipient mice was achieved by gavage with 200 μl of the supernatant from the fecal sample once a day for 8 weeks^11^.

Every mouse was weighed once a week, and weekly food consumption per mouse for the different groups was calculated. Mice were sacrificed after 16 weeks, and fasting blood from heart was collected. Liver tissue, small intestine, cecal contents and epididymal fat tissue from each mouse were either fixed in 4% paraformaldehyde solution, frozen in optimal cutting temperature gel, or snap frozen in liquid nitrogen and stored at −80 °C.

### Microbial analysis of the feces

Fecal samples were collected immediately upon defecation from each mouse at baseline (1^th^ week) and at 16^th^ week and stored at −80 °C. Fecal DNA was extracted from fecal samples using the E.Z.N.A Soil DNA Kit (Omega Bio-tek, Norcross, GA, U.S.) according to the manufacturer’s protocols. The V4-V5 region of the bacteria 16 S ribosomal RNA gene was amplified by PCR. Amplicons were extracted from 2% agarose gels and purified using the AxyPrep DNA Gel Extraction Kit (Axygen Biosciences, Union City, CA, U.S.) according to the manufacturer’s instructions and quantified using QuantiFluor™ -ST (Promega, U.S.). Purified amplicons were pooled at equimolar concentrations and paired-end sequenced (2 × 250) on an Illumina MiSeq platform according to standard protocols. Processing of the sequencing data and bioinformatics analysis were conducted by Majorbio in Shanghai^12^. These sequences were clustered into operational taxonomic units (OTUs) with a 97% sequence identity using mothur (furthest neighbor method) and chopseq (Majorbio). Rarefaction analysis was performed using mothur and plot-rarefaction (Majorbio). From these analyses, the Shannon diversities and Chao1 richness estimations were calculated using mothur. The unweighted UniFrac distance was used to quantify differences in community composition. Principal coordinates analysis (PCoA) was generated by using the R package vegan to demonstrate the clustering of different samples (Majorbio). Nonmetric multidimensional scaling (NMDS) diagrams^13^ were generated using the R package vegan to demonstrate the clustering of different samples. The hierarchical cluster analysis was performed using MVSP 3.1 software (Majorbio)^14^. The linear discriminant analysis effect size (LEfSe) was used to select OTUs that exhibited significance in structural segregation among the grouping of samples (Majorbio)^15^.

### Serum assays

Fasting serum alanine aminotransferase (ALT), aspartate aminotransferase (AST) and blood glucose (FBG) were measured using an automated analyzer (Sysmex CHEMIX-180, Japan). Serum insulin (Rat/Mouse Insulin ELISA Kit, Merck-Millipore) was measured by an enzyme-linked immunosorbent assay, mouse endotoxin concentration in serum was measured by enzyme-linked immunosorbent assay (Mouse ET ELISA Kit, Trust Specialty Zeal), and samples and standards were processed according to the manufacturer’s instructions. The homeostasis model assessment of insulin resistance (HOMA-IR) was calculated by the equation: FBG (mmol/L) × insulin (mU/L)/22.5. The insulin sensitivity index (ISI) was calculated by the equation: 1/ (FPG (mmol/L) × insulin (mU/L)).

### Triglyceride and cholesterol measurements in liver

Intrahepatic triglycerides (TG) and cholesterol were measured by a triglyceride assay kit or cholesterol assay kit (Applygen Technologies Inc., Beijing, China). Samples and standards were then processed according to the manufacturer’s instructions. The final concentrations of triglycerides and cholesterol were corrected for protein content.

### Histological analysis

Paraformaldehyde-fixed paraffin sections of the liver and small intestine were stained with hematoxylin–eosin for pathological analysis or with Masson’s trichrome stain for fibrosis. The non-alcoholic fatty liver activity score (NAS) was assessed and frozen sections of liver were stained with oil red O. For immumohistochemical staining, paraffin-embedded sections were used. Horseradish peroxidase-conjugated secondary antibody was applied and the reaction was visualized by 3, 3′-diaminobenzidine tetrahydrochloride. Slides were counterstained with hematoxylin. Positive areas were quantified with Image J2x. Anti-Foxp3 and anti-ZO-1 antibodies were purchased from Abcam(MA, USA), and IFN-γ, IL-4, IL-17 and IL-22 were purchased from Bioworld(MN, USA).

### Real-time quantitative polymerase chain reaction (qPCR)

Total RNA was extracted from the liver, small intestine or epididymal fat using Trizol (D9108B, Takara, Dalian, China) and was reverse-transcribed into cDNA using Primescript RT Master Mix (RR036A, Takara, Dalian, China). Real-time qPCR was performed on an Applied Biosystems 7500 Real-time PCR system using the SYBR Premix Ex Taq (Tli RnaseH Plus) (RR420A, Takara, Dalian, China). Primers for the target genes were synthesized by Sangon Biotech (Shanghai, China). Primer sequences for genes are listed in Supplementary Table 1. Primer specificity was confirmed by a dissociation curve using the 7500 system SDS software. Glyceraldehyde 3-phosphate dehydrogenase (GAPDH) was used as the internal control.

### Western blot analysis

Livers were lysed in ice-cold radio-immunoprecipitation assay (RIPA) buffer containing protease and phosphatase inhibitors (Phenylmethylsulfonyl fluoride, PMSF) (Beyotime, Shanghai, China). Total protein was measured by the bicinchoninic acid protein assay (BCA, Beyotime, Shanghai, China) method. Foxp3, IFN-γ, IL-4, IL-17, IL-22 and insulin receptor (IR, Abcam) in the liver were detected and actin was used as a loading control. Immune complexes were detected using immobilon western chemiluminescent HRP substrate (Millipore Corporation, Billerica, MA). Bands were quantified by Image Lab Version 2.0.1 (Bio-Rad, Hercules, CA).

### Short chain fatty acids quantification of cecal contents

To determine the level of SCFAs, analytic High Pressure Liquid Chromatography (HPLC, Agilent 1200, Wilmington, DE, USA) was performed. In brief, standard solutions of acetate, propionate and butyrate (all from Sigma-Aldrich) were prepared at various concentrations (3–6000 ng/ml). These solutions were analyzed using HPLC, and cecal contents were dissolved in 0.1% formic acid and analyzed in a similar manner to measure the total concentration of all three free fatty acids^11^.

### Statistical analysis

Results are presented as the mean with the standard error of mean (SEM). Graph Pad prism (version 6.01) was used to conduct all statistical tests. Comparisons were performed using a one-way analysis of variance test (ANOVA) and Post hoc Student–Newman–Kuels analyses were performed when >2 groups. Results were considered statistically significant when *p* < 0.05.

## Results

### Effects of FMT on body weight, HOMA-IR and serum transaminases

**Figure 1:**
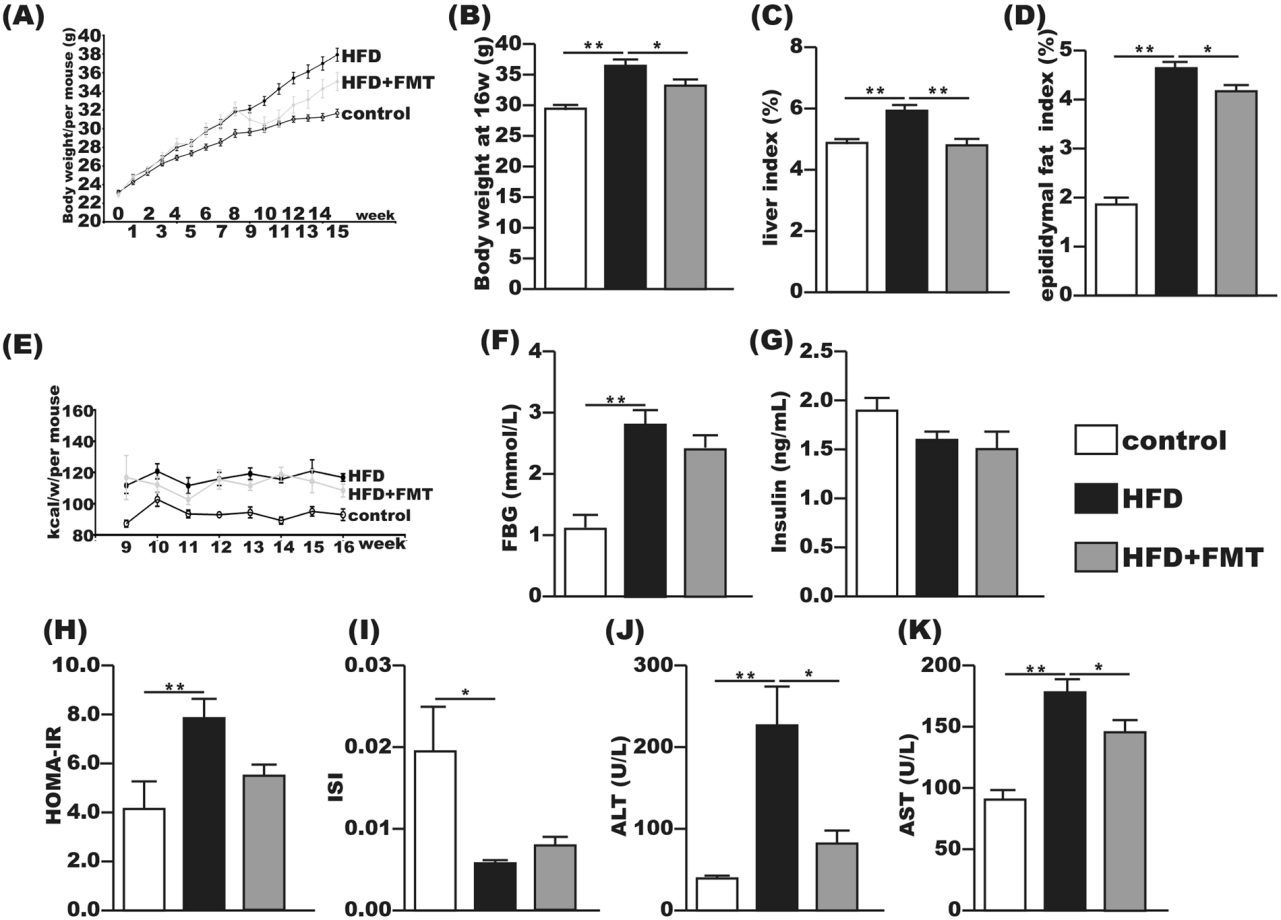
FMT attenuated HFD-induced obesity, liver injury and metabolic disturbance. (**A,B**) Body weight changes and body weight at 16th week. (**C**) Liver index = liver weight/body weight × 100. (**D**) Epididymal fat index = epididymal fat weight/body weight × 100. (**E**) Energy intake per mouse per week. (**F,I**) Fasting serum glucose, fasting serum insulin, HOMA-IR and ISI of the three groups. **J,K**) Serum ALT and AST. The data represent the mean ± S.E.M. (n = 12 mice per group), ^*^ *P* < 0.05, ^**^ *P* < 0.01 and ^***^ *P* < 0.001.

After 16 weeks of the experiment, mice in the HFD group gained more body weight than the control group. The liver index and epididymal fat index were significantly increased by 20% and 125%, respectively, compared with the control group. After 8 weeks of FMT intervention, the body weight, liver index and epididymal fat index in the HFD + FMT group were significantly decreased compared to those in the HFD group (Fig. 1A–D). However, weekly energy intake showed no significant difference between the HFD and HFD + FMT groups (Fig. 1E). FBG was significantly elevated in the HFD group compared with the control group, but exhibited no significant difference between the HFD and HFD + FMT group (Fig. 1F). The serum insulin level was not significantly changed among the three groups (Fig. 1G). The HOMA-IR was increased and the ISI was decreased in the HFD group compared with those of the control group, but the HOMA-IR and ISI were not significantly improved in the HFD + FMT group when compared with the HFD group (Fig. 1H,I). The serum levels of ALT and AST were significantly increased in the HFD group compared with the control group and significantly decreased after the 8-week FMT intervention (Fig. 1J,K).

### Improvement of FMT on the gut microbiota and increase in butyrate concentrations of the cecal contents

**Figure 2:**
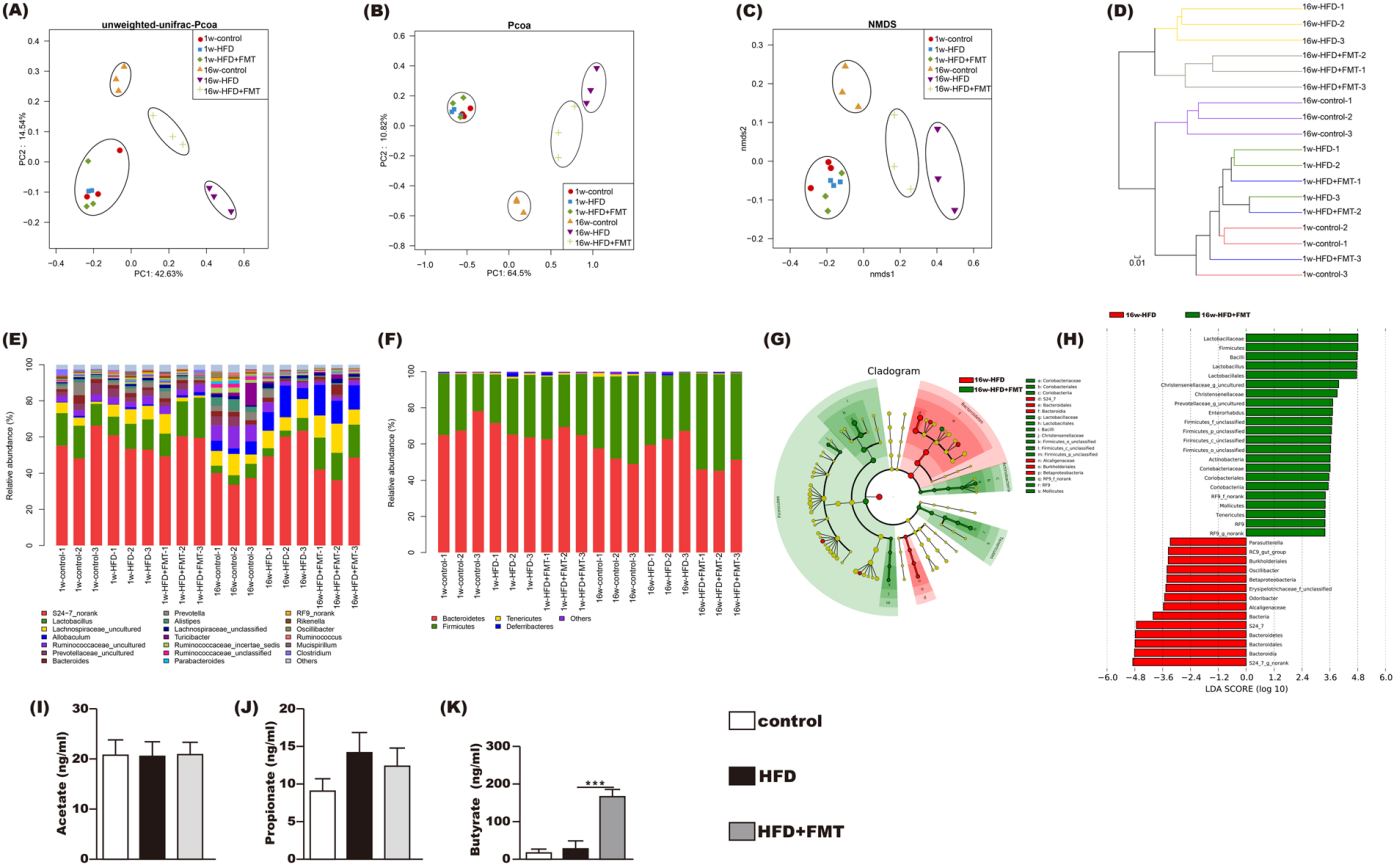
Influences of FMT on gut microbiota. (**A,B**) Scatter plot of the unweighted-unifrac-principal co-ordinates analysis (PCOA) score showing the similarity of the 18 bacterial communities based on the Unifrac distance and Scatter plot of the weighted-PCOA score. (**C**) Nonmetric multidimensional scaling (NMDS) showing the difference in bacterial communities according to the Bray-Curtis distance. **(D)** Hierarchical cluster analysis. (**E,F**) Bacterial composition of the different communities at the genus level (**E**) and at phylum level (**F**). Sequences that could not be classified into any known group were assigned as “no rank.” (**G,H**) Differences were represented in the color of the most abundant group. Key phylotypes of the gut microbiota responding to FMT treatment (**G**), the histogram (**H**) showing the lineages with LDA values as determined by LEfSe. (**I,K**) The acetic acid, propanoic acid and butyrate acid levels of the cecal content. The data represent the mean ± S.E.M. (n = 12 mice per group), ^***^ *P* < 0.001.

To reveal the effects of HFD and FMT on the microbiota structure, we sequenced the bacterial 16S rRNA at baseline and after 16 weeks. Unweighted PCoA, PCoA and NMDS analyses were performed to provide an overview of the gut microbiota composition of the six animal groups at baseline and at the end of the trial. The plotted scores indicated no detectable difference in the microbiota composition among the groups before the intervention but clearly separated the 16w-HFD mice from the 16w-control mice, with the 16w-HFD + FMT group distributed in between. This indicated that the FMT intervention shifted the overall structure of the HFD-disrupted gut microbiota toward that of the control mice (Fig. 2A–C). Furthermore, the hierarchical cluster analysis showed that the 1w-control, 1w-HFD, 1w-HFD + FMT, and the 16w-control communities grouped together and then clustered with the 16w-HFD + FMT and 16w-HFD communities in order (Fig. 2D).

Sixteen weeks of HFD feeding induced widespread changes in the gut microbial community structure at the phylum level compared with the control. There was an increase in the abundance of Bacteroidetes (63.1% *vs*. 52.9%) and decreases in the abundances of Actinobacteria (0.04% *vs*. 0.15%) and Firmicutes (35.8% *vs*. 44.6%). However, FMT intervention mitigated the HFD-induced decrease in Actinobacteria and Firmicutes, and the HFD-induced increase in Bacteroidetes. At the genus level, *Lactobacillus*, *Christensenellaceae_uncultured*, *Prevotellaceae_uncultured* were decreased in the HFD group compared to the control, all of which were reversed by FMT intervention. FMT intervention decreased the abundances of *Odoribacter* and *Oscillibacter* compared with the HFD group (Fig. 2E,F). LefSe analysis also showed that the FMT intervention was associated with changes at the genus level (Fig. 2G,H).

The concentrations of acetate and propionate in the cecal content showed no differences among the three groups (Fig. 2I,J). The concentration of butyrate in cecal contents showed no difference between the control and HFD group but was significantly increased in the HFD + FMT group (Fig. 2K).

### FMT intervention improved the tight junction and morphometry of the small intestine and endotoxemia

**Figure 3:**
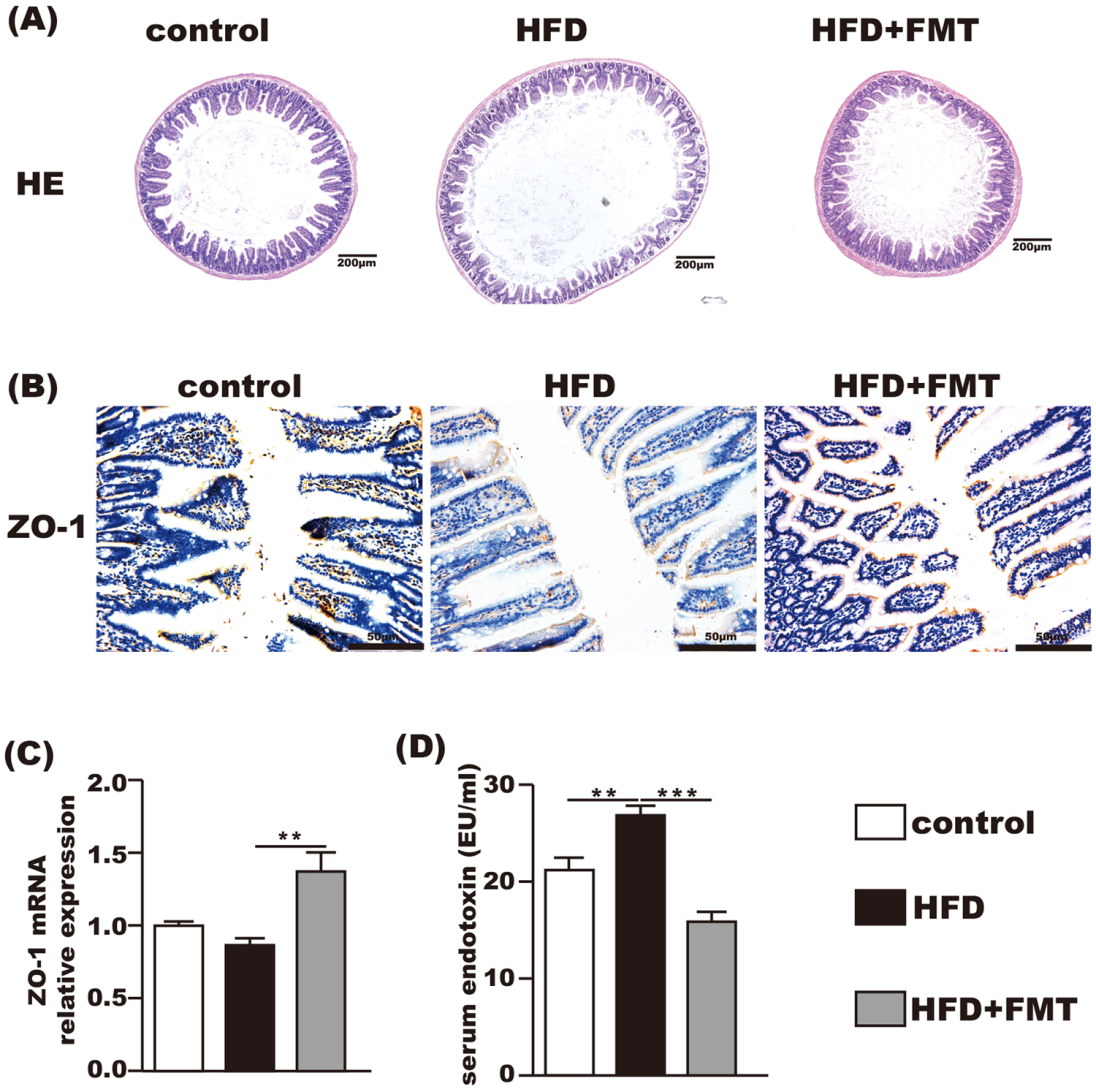
Beneficial effects of FMT on the small intestine and the level of serum endotoxin. (**A**) HE staining of the small intestine showing that FMT restored HFD-induced mucosal damage. (**B**) Immunohistochemistry for ZO-1 showing that FMT increased ZO-1 expression. **(C)** ZO-1 mRNA expression in the small intestine. (**D**) The level of serum endotoxin. The data represent the mean ± S.E.M. (n = 12 mice per group), ^*^ *P* < 0.05, ^**^ *P* < 0.01 and ^***^ *P* < 0.001.

The HE staining showed that the HFD-induced intestinal mucosa injury was alleviated by FMT intervention (Fig. 3A). Immunohistochemistry and qPCR demonstrated that the expression of ZO-1 in the small intestine was significantly increased after FMT intervention compared with the HFD group (Fig. 3B,C). In addition, the serum endotoxin level was significantly elevated in the HFD group but was decreased after FMT intervention (Fig. 3D).

### FMT treatment attenuated HFD-induced steatohepatitis

**Figure 4:**
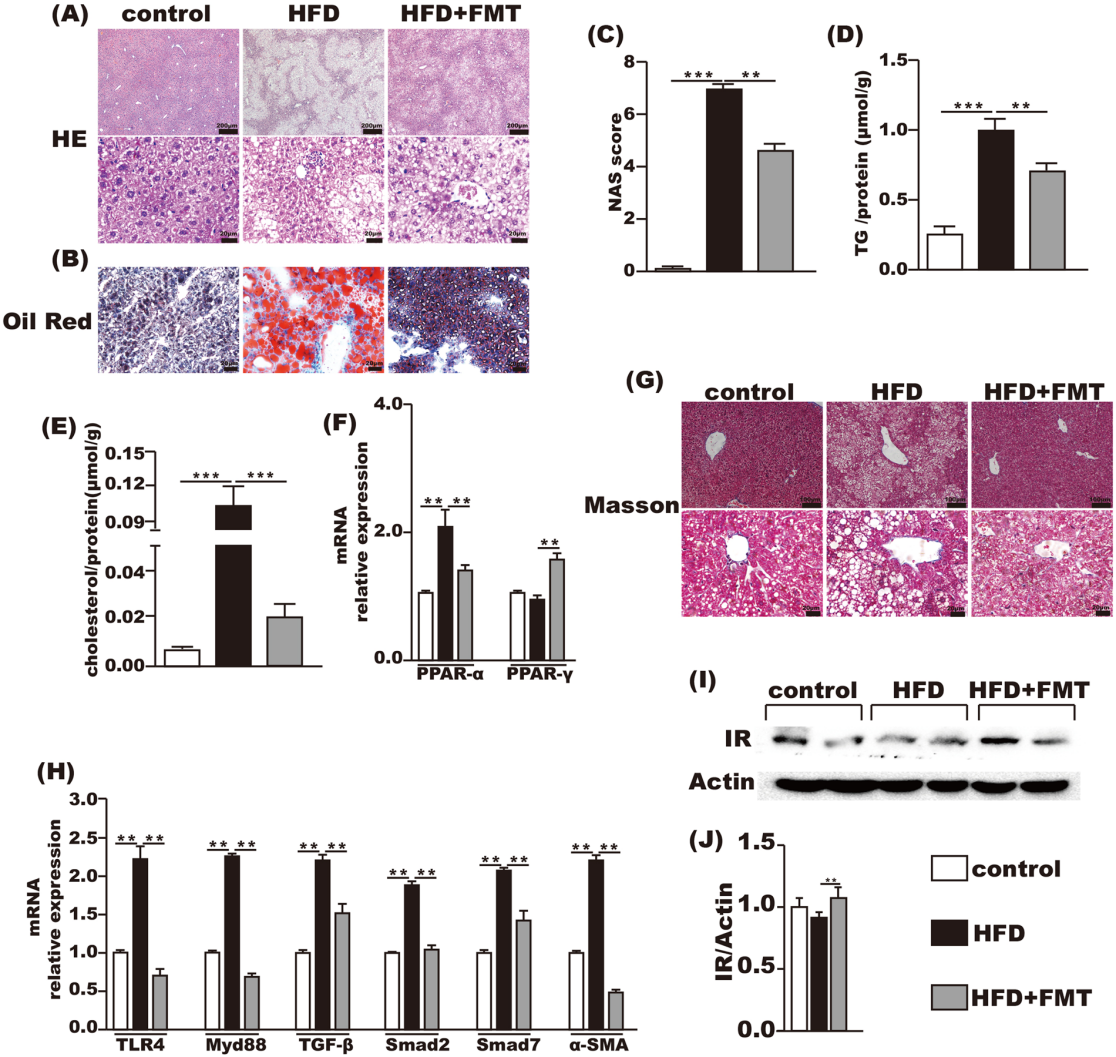
FMT improved inflammation and lipid metabolism in liver. (**A,B**) HE and oil red O staining of liver. (**C**) NAS score. (**D,E**) the level of TG and cholesterol in the liver. (**F**) Lipid metabolism-associated PPAR-α and PPAR-γ gene expression in the liver. (**G**) Masson’s staining. (**H**) Endotoxin-associated gene expression of TLR4 and Myd88 and fibrosis-associated gene expression of TGF-β, Smad2, Smad7 and α-SMA. (**I,J**) IR protein expression level in liver. Gene expression levels are expressed as values relative to the control group. The data represent the mean ± S.E.M. (n = 12 mice per group), ^*^ *P* < 0.05, ^**^ *P* < 0.01 and ^***^ *P* < 0.001.

The incidence of steatohepatitis induced by chronic feeding with HFD was 100% in the HFD group. The liver HE staining showed that the control group had no lipid accumulation, and marked lipid accumulation was observed in the HFD group, which was significantly attenuated by FMT treatment (Fig. 4A). This result was further confirmed by oil red O staining (Fig. 4B). The average NAS score of the liver was 6.90 ± 0.233 in the HFD group and was decreased to 4.58 ± 0.260 after FMT intervention (Fig. 4C). Intrahepatic TG and cholesterol contents were increased by approximately 4-fold and 17-fold, respectively, in the HFD group when compared with the control group. This increase was significantly reduced after FMT intervention (Fig. 4D,E). PPAR-α mRNA in the liver increased 2-fold after HFD when compared with the control group but was decreased by FMT intervention. There was no difference in hepatic PPAR-γ mRNA between the HFD and control groups, but it was significantly increased in the HFD + FMT group (Fig. 4F).

Although Masson’s staining of the liver exhibited no fibrosis in all the three groups (Fig. 4G), the mRNA levels of fibrosis-associated genes in the liver, such as TGF-β1, α-SMA, Smad7 and Smad2, were increased two times more in the HFD group than the control group, whereas FMT intervention reduced the expression of these genes in the liver (Fig. 4H). Hepatic TLR4 and Myd88 mRNA were also significantly increased in the HFD group compared with the control group and were suppressed by FMT intervention (Fig. 4H). And the protein level of insulin receptor in the liver was significantly increased after FMT intervention compared with the HFD group (Fig. 4I,J).

### Effect of FMT on the intrahepatic immune

**Figure 5:**
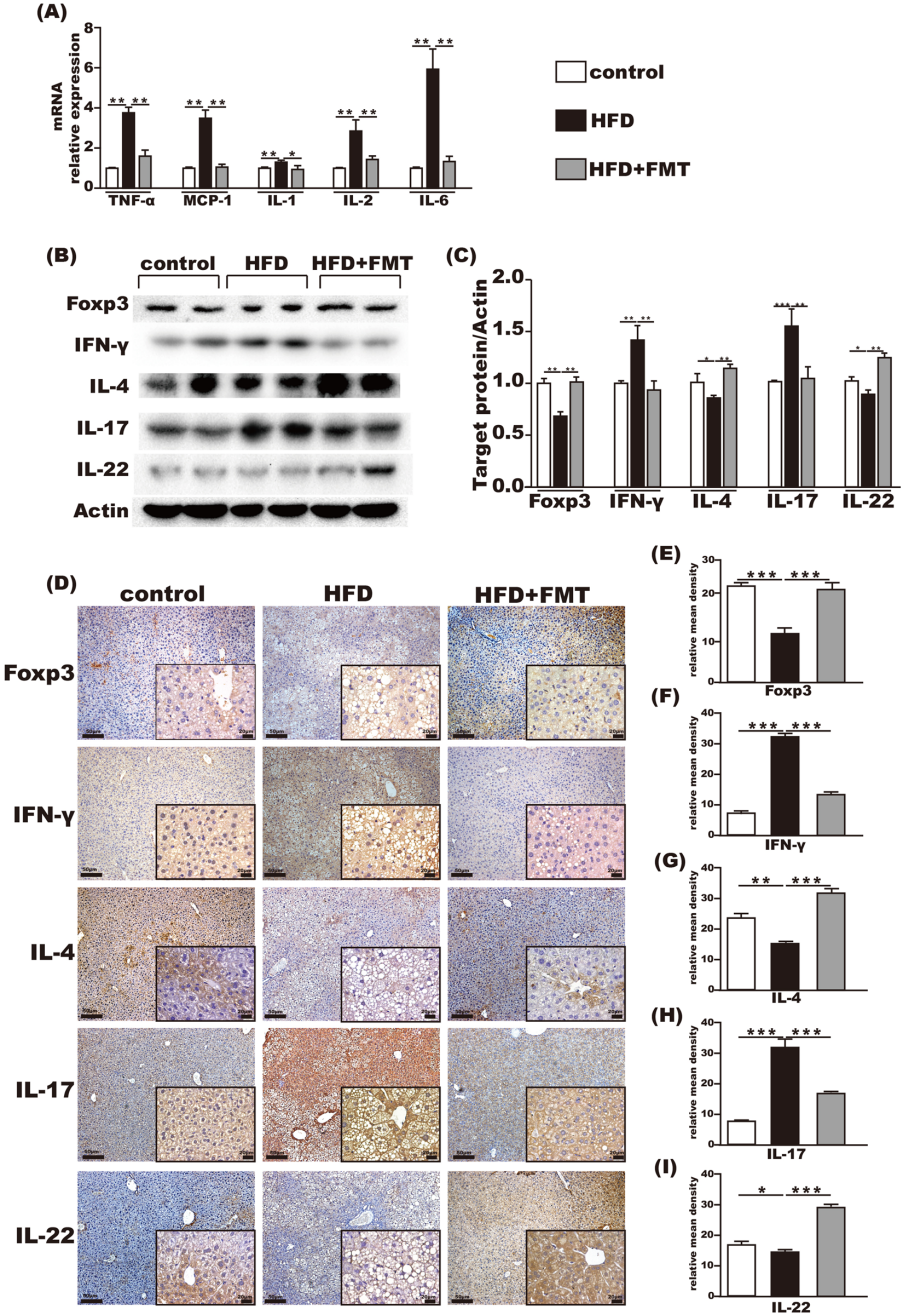
Immunoregulation of FMT in the liver. (**A**) Pro-inflammation-associated gene expression in the liver. (**B,C**) Foxp3, IFN-γ, IL-4, IL-17 and IL-22 protein expression levels in the liver. (**D–I**) Immunohistochemistry for Foxp3, IFN-γ, IL-4, IL-17 and IL-22 in the liver showed that Foxp3, IL-4, and IL-22 were increased and IFN-γ and IL-17 were decreased after FMT intervention. The data represent the mean ± S.E.M. ^*^ *P* < 0.05, ^**^ *P* < 0.01 and ^***^ *P* < 0.001.

The mRNA levels of TNF-α, MCP-1, IL-1β, IL-2 and IL-6 in the liver were upregulated in the HFD group compared with the control group, all of which were reduced by FMT intervention (Fig. 5A).

Compared with the control group, hepatic protein levels of Foxp3, IL-4 and IL-22 in the HFD group were significantly decreased and the expressions of IFN-γ and IL-17 were significantly increased. FMT intervention effectively reversed these imbalanced immune factors, which increased the levels of Foxp3, IL-4 and IL-22 and decreased IFN-γ and IL-17 in the liver (Fig. 5B,C).

Expressions of Foxp3, IFN-γ, IL-4, IL-17 and IL-22 in the liver were also detected by immumohistochemical staining. The results showed that Foxp3, IL-4 and IL-22 were decreased, whereas IFN-γ and IL-17 were relatively increased in the HFD group compared with the control group. This was reversed by FMT intervention (Fig. 5D–I).

### Effect of FMT on the epididymal fat tissue

**Figure 6:**
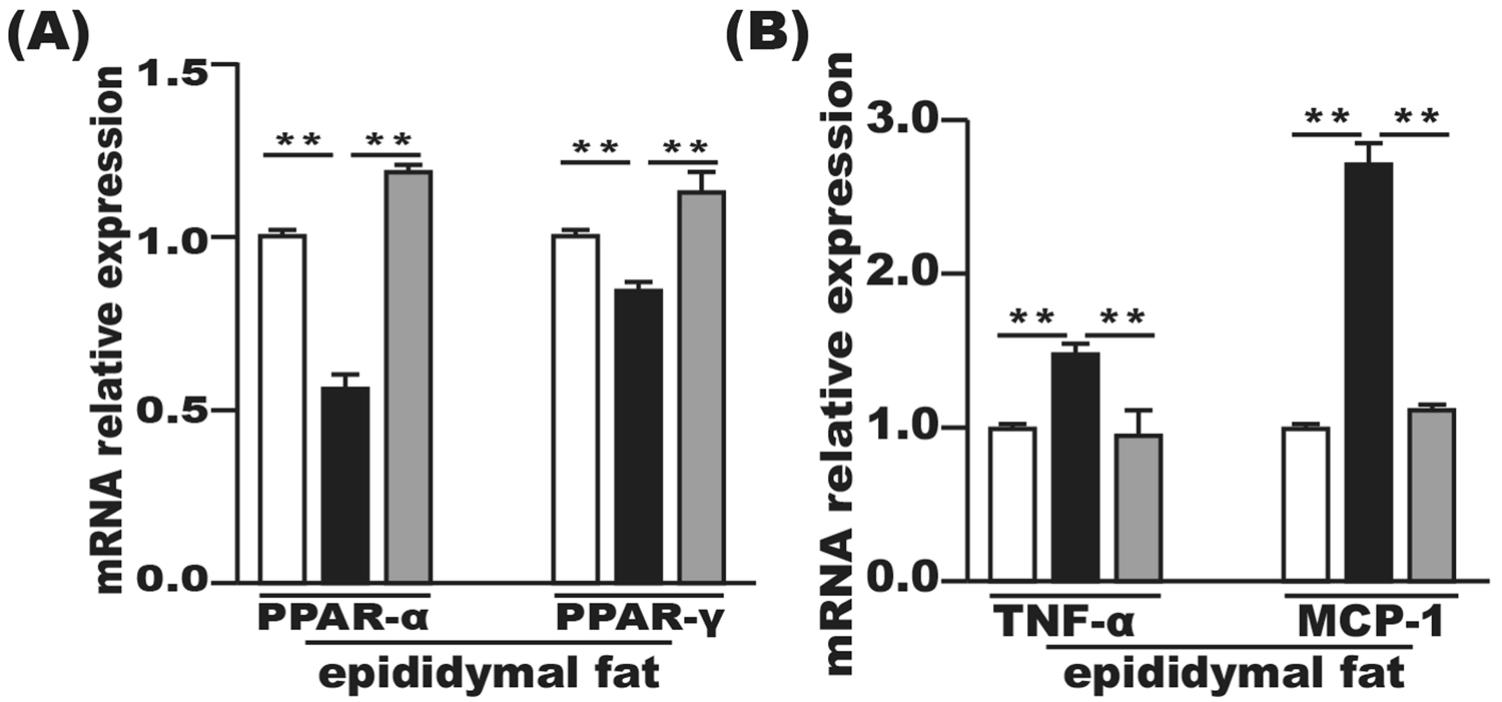
FMT improved inflammation and lipid metabolism in epididymal fat tissue. **(A)** Lipid metabolism-associated PPAR-α and PPAR-γ gene expression in the epididymal fat tissue. (**B**) Gene expression levels of MCP-1 and TNF-α in the epididymal fat tissue. Gene expression levels are expressed as values relative to the control group. The data represent the mean ± S.E.M. (n = 12 mice per group), ^*^ *P* < 0.05, ^**^ *P* < 0.01 and ^***^ *P* < 0.001.

The PPAR-α and PPAR-γ mRNAs in the epididymal fat tissue were significantly downregulated in the HFD group compared with the control group and significantly upregulated after FMT intervention (Fig. 6A). Furthermore, the mRNA levels of TNF-α and MCP-1 in the epididymal fat tissue were increased in the HFD group compared with the control group, which were suppressed by FMT intervention (Fig. 6B).

## Discussion

In this study, we demonstrated that the gut microbiota and intrahepatic cytokine profiles in mice were significantly disturbed by 16-week HFD. However, 8-week FMT intervention corrected the gut microbiota disturbance to a certain degree and increased the production of butyrate in the cecal content along with the correction of the imbalance of pro- and anti-inflammatory cytokines and the reversion of steatohepatitis in mice chronic fed with HFD. These results indicated that FMT may have profound implications in the treatment of NASH.

With NASH becoming more and more prevalent worldwide, it is urgent to find an economical and effective therapeutic approach to prevent the onset and progression of NASH. Rapidly increasing evidence supports the association between NASH and the gut microbiota^16^. Meanwhile, FMT has emerged as a highly effective treatment for some gastrointestinal diseases such as gastrointestinal infections and inflammatory bowel disease. There is a growing number of studies on the intestinal microbiota or FMT in a wide range of non-gastrointestinal diseases, including metabolic disorders^5, 17^. A previous clinical study conducted by Vrieze showed that the insulin sensitivity of recipients increased along with the levels of butyrate-producing intestinal microbiota^18^. Although the internal mechanisms are still unknown, this offers a rationale for novel therapeutic interventions for metabolic diseases, including NASH. In our study, we found that after 8 weeks of FMT intervention, the metabolism-related indicators, such as body weight, liver index, and epididymal fat index, were obviously improved, with a tendency toward improvement in FBG, HOMA-IR and ISI. This may suggest a beneficial effect of FMT in HFD-induced metabolic disturbances. Furthermore, serum levels of transaminases, and the degree of hepatic steatosis, lobular inflammation and hepatocyte ballooning were markedly attenuated after FMT intervention.

To elucidate the underlying mechanisms, we detected the gut microbiota of the different groups at baseline and the 16^th^ week. According to the PCoA, NMDS and the hierarchical cluster analysis, FMT reversed the overall changes of the gut microbiota structure induced by HFD, and the HFD + FMT were distributed between the HFD and control groups at the 16^th^ week. At the phylum level, the Bacteroidetes phylum was decreased and the Firmicutes phylum was increased after FMT intervention compared with the HFD group. Although the Firmicutes/Bacteroidetes ratio was still inconsistent in patients with NAFLD^2^, bacteria from the Firmicutes phylum could efficiently ferment fiber into butyrate, while bacteria from the Bacteroidetes phylum were involved in fermenting fiber into acetate and propionates. I^19^. Our results showed that proportion of *Lactobacillus* from Firmicutes phylum and butyrate level in the gut was both significantly elevated after FMT intervention. This result is supported by the finding that the Bacterial strains from *Lactobacillus* could increase the proportions of butyrate-producing bacterial strains^20^, and that lactate from *Lactobacillus* could promote butyrate production in feces^21^. Nonetheless, acetate and propionate were not significantly decreased in mice on HFD following a slight decrease in Bacteroidetes. The underlying mechanism was not clear in current study. The fermentation process to produce short-chain fatty acids in the gut is rather complex under different conditions. Bacteroidetes could ferment fiber into acetate and propionate, besides, there are other pathways involved in production of acetate and propionate^22^. Based on those backgrounds, we speculate that a slight decrease in Bacteroidetes as we observed here may not necessarily lead to a significant decrease in the basic levels of acetate and propionate in the gut, since they are affected by multiple other factors. And Butyrate has multiple beneficial effects on mammals. Previous studies showed that butyrate protected the intestinal barrier function and maintained the integral bifidogenic effect^23, 24^, which is fundamental in the maintenance of gut health. Our results showed that the tight junction protein ZO-1 was also significantly upregulated after FMT intervention along with improved intestinal morphometry and decreased levels of serum endotoxin. Increased intestinal permeability and metabolic endotoxemia following deleterious composition of the intestinal microbiota contributes to the progression of NASH^5^. The endotoxin is derived from the gram-negative bacteria. The relative abundance of gram-negative, putative “pro-inflammatory” bacteria in various family and genus level taxa from the phylum Bacteroidetes were also decreased after FMT intervention compared with other groups. Decrease in gram-negative bacteria might contribute to reduced production of endotoxin in the gut, which may be partially responsible for decreased endotoxin level in the FMT group than the healthy controls as we observed here^25^. In addition, the proportion of *Lactobacillus* was elevated the most after FMT intervention. *Lactobacillus* is a well-known probiotic with multiple beneficial effects on body metabolism. *Lactobacillus* could decrease the endotoxin level, inhibit endotoxin-induced inflammation and regulate immunity. Increase of Lactobacillus after FMT intervention might further reduce opportunistic pathogens and endotoxin level^26–29^. Alleviation of endotoxemia was further supported by the decreased expression of TLR4 and its downstream signaling protein Myd88 in the liver after FMT intervention. These results suggest that FMT might attenuate HFD-induced NASH via restoring intestinal health. FMT intervention significantly decreased the TGF-β1 and α-SMA level, to a level even lower than those of the control group. There are several explanations for this finding. First, TLR4 plays a pivotal role in the pro-inflammatory response and liver fibrosis, the downregulation of TLR4 signaling pathway following decreased endotoxin after FMT would lead to the inhibition of TGF-β1 and α-SMA expression^30^. Second, others showed that NaB could bring the expression of TGF-β1 and α-SMA down to a level lower than those of the control group via inhibiting histone deacetylases^31, 32^. In the current study, NaB was significantly elevated in HFD + FMT group compared with that of the control group and HFD group. When comparing the taxonomy profile at the genus level, *Lactobacillus* and *Christensenellaceae* were increased in the HFD + FMT group, and this increase may be related to the bifidogenic effect of FMT via the fermentation product butyrate^33^. A previous study showed that *Christensenellaceae* is positively associated with a low body mass index in humans and reduced weight gain in mice^34^. *Oscillibacter* was increased in the HFD group but decreased after FMT intervention at the present study. This finding is consistent with another study that shows a positive and strong association of *Oscillibacter* with metabolic syndrome and the development of obesity-related metabolic disorders^35, 36^. Although the mechanisms of interaction between these specific gut microbes and the host metabolism still need further exploration, our results suggest that the modulation of the gut microbiota might be an effective strategy for managing NASH.

Gut microbiota-host immune maladaptation has been implicated in the rising incidence of NASH. The progression of NASH is greatly associated with the impaired immune microenvironment^37, 38^. The gut microbiota metabolite butyrate, which obviously increased after FMT intervention, is a pivotal immune regulator and anti-inflammatory substance. It improves the hepatic insulin resistance and attenuates HFD-induced steatohepatitis^23, 39–41^. In this study, the pro-inflammatory factors in the liver, such as TNF-α, MCP-1, IL-1 and IL-6, were decreased in the FMT-treated mice. This change would favor an anti-inflammatory immune microenvironment in the liver. Accordingly, the TLR4-Myd88 pathway, which was also depressed by FMT, could aggravate the development of insulin resistance^42^. In addition, PPAR-α, a gene that mainly participates in fatty acid oxidation, and PPAR-γ, a gene that plays pivotal roles in the regulation of lipid metabolism and inflammation as well as in insulin sensitivity^42, 43^, were both increased in the liver and epididymal fat tissue, while TG and cholesterol were decreased in the liver after FMT intervention. And hepatic steatosis was attenuated in the absent of improved serum fasting glucose, insulin level or HOMA-IR. The improvement in hepatic steatosis may result from enhanced intrahepatic insulin sensitivity after FMT. Others showed that in mice with NASH, supplementation of sodium butyrate caused increased expression of insulin receptor in the liver, leading to improved liver insulin sensitivity and hepatic steatosis^41^. Here we also showed that in mice subjected to FMT, the protein level of insulin receptor was upregulated, following a significant increase of butyrate concentration in cecal content after FMT. These findings warrant further studies to reveal other factors and mechanisms in enhancing intrahepatic insulin sensitivity after FMT.

Different subsets of T cells are involved in the progression of NASH. These cells contribute to an impaired liver immune microenvironment. Butyrate has the ability to regulate T cell differentiation^23, 40^, which suggests that FMT might correct the ratios of T cell subsets via the gut metabolite butyrate. Our results showed that IFN-γ (Th1) and IL-17 (Th17) were decreased and IL-4 (Th2), IL-22 (Th22) and Foxp3 (Treg) were increased by FMT in HFD-fed mice. These changes may result in a preferred T cell subset^38, 44–46^. Existing evidence revealed that the reduction of Treg cells in the liver could promote the transformation of simple hepatic steatosis to steatohepatitis. Animal experiments indicated that adoptive transfer of Treg cells into mice could alleviate HFD-induced steatohepatitis^38, 47^. Cytokines such as IFN-γ and IL-17 from Th1 and Th17 cells can promote the release of IL-6 and TNF-α, which aggravate liver insulin resistance and steatohepatitis^46, 48^. Previous work showed that intrahepatic IL-17 was significantly elevated in NASH patients. IL-17 could inhibit the insulin-signaling pathway and aggravate hepatocyte steatosis and the extent of liver inflammation^49, 50^. IL-22 is known to be a hepatocyte protector that could inhibit liver steatosis and liver damage^45, 51^. All the above research supports the point that FMT might be beneficial in immunologic balance of the liver.

Our study has the following limitations. First, we did not perform a metabolomic analysis of the cecal content. This analysis would be helpful to elucidate the influences of FMT on mice. Second, we only conducted the 16s rRNA sequencing of the gut microbiota. Whole genome sequencing would be more precise for finding the internal interactions from the perspective of gene function.

In summary, our results showed that FMT attenuated HFD-induced steatohepatitis in mice. until now, patients who undergo FMT are mainly via endoscopy, so it is impossible to conduct FMT on a high frequency. Our result supports a possible way of oral FMT through more economic and convenient methods such as feces capsules, which is under investigation in human. Development of microbiome-targeted therapeutic strategies should be considered to open the door to new ways of prevention and treatment of NASH.

## Electronic supplementary material

Supplementary Information
