# Supplementary material for: Total fecal microbiota transplantation alleviates high-fat diet-induced steatohepatitis in mice via beneficial regulation of gut microbiota
Source: Sci Rep. 2017 May 8;7:1529. doi: 10.1038/s41598-017-01751-y (PMC5431549; doi:10.1038/s41598-017-01751-y)
Supplement: Supplementary file 1 — Supplementary Information [file 41598_2017_1751_MOESM1_ESM.pdf]

Total fecal microbiota transplantation alleviates high-fat diet-induced steatohepatitis in mice via beneficial regulation of gut microbiota

Da Zhou[1#], Qin Pan[1#], Feng Shen[1], Hai-xia Cao[1], Wen-jin Ding[1], Yuan-wen Chen[1]*, Jian-gao Fan[1]*

[1] Center for Fatty Liver, Department of Gastroenterology, Xinhua Hospital Affiliated to Shanghai Jiao Tong University School of Medicine, Shanghai, 200092, China

***Corresponding author**

Professor Jian-gao Fan, Email: fattyliver2004@126.com;

Dr. Yuan-wen Chen, Email: shsmus@263.net.

[#]Da Zhou and Qin Pan contributed equally to this work.

Supplementary Table S1. Real-time PCR primers

Gene	Forward primer	Reverse primer
ZO-1	5’-CTCCAGGTGCTTCTCTTGCT-3’	5’-TATCTTCGGGTGGCTTCACT-3’
PPAR-α	5’-TGGTTGAATCGTGAGGAACA-3’	5’-ATCGCCACTAAGGTGTCAGG-3’
PPAR-γ	5’-CAGGAGCAGAGCAAAGAGGT-3’	5’-TGGACACCATACTTGAGCAGA-3’
TGF-β1	5’-ATTCCTGGCGTTACCTTGG-3’	5’-AGCCCTGTATTCCGTCTCCT-3’
α-SMA	5’-AGGGAGTAATGGTTGGAATGG-3’	5’-GGTGATGATGCCGTGTTCTA-3’
Smad7	5’-CAAGAGGCTGTGTTGCTGTG-3’	5’-TGGGTATCTGGAGTAAGGAGGA-3’
Smad2	5’-CGTCCATCTTGCCATTCAC-3’	5’-TCCTGTCCATTCTGCTCTCC-3’
TNF-α	5’-AAGGGAGAGTGGTCAGGTTG-3’	5’-TCTGTGAGGAAGGCTGTGC-3’
MCP-1	5’-CCTGCTGCTACTCATTCACC-3’	5’-GGACCCATTCCTTCTTGG-3’
IL-1β	5’-TGGGCTGGACTGTTTCTAATG-3’	5’-GGTTTCTTGTGACCCTGAGC-3’
IL-2	5’-CGGCATGTTCTGGATTTGAC-3’	5’-CATCATCGAATTGGCACTCA-3’
IL-6	5’-CGGAGAGGAGACTTCACAGAG-3’	5’- ATTTCCACGATTTCCCAGAG-3’
TLR4	5’-TCAGAGCCGTTGGTGTATCTT-3’	5’-CCTCAGCAGGGACTTCTCAA-3’
Myd88	5’-GCCTTGTTAGACCGTGAGGA-3’	5’-GGGACACTGCTTTCCACTCT-3’
